# AgroSeek: a system for computational analysis of environmental metagenomic data and associated metadata

**DOI:** 10.1186/s12859-021-04035-5

**Published:** 2021-03-10

**Authors:** Xiao Liang, Kyle Akers, Ishi Keenum, Lauren Wind, Suraj Gupta, Chaoqi Chen, Reem Aldaihani, Amy Pruden, Liqing Zhang, Katharine F. Knowlton, Kang Xia, Lenwood S. Heath

**Affiliations:** 1 Department of Computer Science, Virginia Polytechnic Institute and State University, 24061 Blacksburg, USA; 2 Department of Civil and Environmental Engineering, Virginia Polytechnic Institute and State University, 24061 Blacksburg, USA; 3 Department of Biological Systems Engineering, Virginia Polytechnic Institute and State University, 24061 Blacksburg, USA; 4 Interdisciplinary PhD Program in Genetics, Bioinformatics, and Computational Biology, Virginia Polytechnic Institute and State University, 24061 Blacksburg, USA; 5 Department of Dairy Science, Virginia Polytechnic Institute and State University, 24061 Blacksburg, USA; 6 School of Plant and Environmental Science, Virginia Polytechnic Institute and State University, 24061 Blacksburg, USA; 7 School of Resource and Environmental Science, Wuhan University, 430072 Wuhan, China

**Keywords:** Metagenomics, Metadata, Web server, Data science, Antibiotic resistance

## Abstract

**Background:**

Metagenomics is gaining attention as a powerful tool for identifying how agricultural management practices influence human and animal health, especially in terms of potential to contribute to the spread of antibiotic resistance. However, the ability to compare the distribution and prevalence of antibiotic resistance genes (ARGs) across multiple studies and environments is currently impossible without a complete re-analysis of published datasets. This challenge must be addressed for metagenomics to realize its potential for helping guide effective policy and practice measures relevant to agricultural ecosystems, for example, identifying critical control points for mitigating the spread of antibiotic resistance.

**Results:**

Here we introduce AgroSeek, a centralized web-based system that provides computational tools for analysis and comparison of metagenomic data sets tailored specifically to researchers and other users in the agricultural sector interested in tracking and mitigating the spread of ARGs. AgroSeek draws from rich, user-provided metagenomic data and metadata to facilitate analysis, comparison, and prediction in a user-friendly fashion. Further, AgroSeek draws from publicly-contributed data sets to provide a point of comparison and context for data analysis. To incorporate metadata into our analysis and comparison procedures, we provide flexible metadata templates, including user-customized metadata attributes to facilitate data sharing, while maintaining the metadata in a comparable fashion for the broader user community and to support large-scale comparative and predictive analysis.

**Conclusion:**

AgroSeek provides an easy-to-use tool for environmental metagenomic analysis and comparison, based on both gene annotations and associated metadata, with this initial demonstration focusing on control of antibiotic resistance in agricultural ecosystems. Agroseek creates a space for metagenomic data sharing and collaboration to assist policy makers, stakeholders, and the public in decision-making. AgroSeek is publicly-available at https://agroseek.cs.vt.edu/.

**Supplementary Information:**

The online version contains supplementary material available at 10.1186/s12859-021-04035-5.

## Background

Antibiotic resistance is a serious human and animal health concern, annually resulting in 35,000 deaths in the US [1] and more than 700,000 deaths globally [2]. Agricultural practices, especially the use of antibiotics in livestock, present numerous concerns with respect to their potential to contribute to the evolution and spread of antibiotic resistance [3–6]. The spread of antibiotic resistance genes (ARGs) to and from pathogens and resident non-pathogenic bacteria via horizontal gene transfer in agricultural environments is of special concern [7, 8]. Understanding how agricultural practices impact microbial communities and the dissemination of ARGs in the environment is vital to identifying effective strategies to mitigate the spread of antibiotic resistance [9, 10].

Microbial communities associated with agricultural ecosystems, including feed, rumen, gut, manure, compost, soil, crops, and livestock products, are vast and diverse and various tools have been applied for their analysis. Given the diversity of these microbial communities, culture and targeted molecular methods only capture the tip of the iceberg of the true reservoir of genetic information (e.g., taxonomic profiles, ARGs, and mobile genetic elements). Shotgun metagenomics takes advantage of next-generation DNA sequencing (NGS) to directly access the full array of genetic material representing a given environmental sample, without selecting desired targets a priori. Metagenomics can be a powerful tool for objectively and comprehensively assessing the effects of various agricultural practices on corresponding microbiomes and resistomes (i.e., total ARGs) of interest. However, comparing conclusions with respect to the composition and response of the resistome across studies has proven to be extremely challenging due to the wide variety of analysis pipelines, differing methods of reporting, and differences in study design [11]. The ability to compare resistomes is vital to establish what is a “normal” baseline pattern/level of antibiotic resistance in the environment versus a potential “red flag” that merits additional attention.

An inherent characteristic of environmental samples in general, and agricultural ecosystem samples in particular, is their sheer complexity and thus the critical nature of associated contextual information needed to accurately evaluate, compare, and predict their corresponding metagenomes. Such “metadata,” (e.g., temperature, pH, farm type, soil type, management practices, DNA extraction method, sequencing platform) will widely vary in composition and format across different environments and studies. Platforms, such as BioProject and BioSample at NCBI, have begun to address this by requiring submission of specific metadata, but these services do not offer data interpretation or comparison [12]. MG-RAST [13] is another publicly-available platform that requires metadata submission for metagenomic analysis. However, the annotation databases incorporated in MG-RAST are not well-tailored for characterizing antibiotic resistance, while the process of uploading metadata is not intuitive or well-adapted to antibiotic resistance studies. Additionally, MetaStorm [14] is an open access user-friendly pipeline that facilitates metagenomic annotation using customized databases. While ARGs can be accurately annotated in MetaStorm, a corresponding metadata repository and analysis tool is lacking, making it impossible to compare across multiple projects. Thus, AgroSeek presents many advantages as a centralized platform specifically tailored to the study of antibiotic resistance in agricultural environments, with a corresponding mechanism to gather relevant metadata in a user-friendly and comprehensive fashion. Gathering such metadata is crucial to the ability to share data across the wider research community, allowing researchers to obtain a general sense of the ranges and distributions of various metagenomic measurements of interest for a given sample or environment type (e.g., feed, manure, soil, water). By revealing the commonalities, differences, and anomalies among various projects contributed by the user community, insights can be gained to reach broader conclusions.

Here, we present AgroSeek, a web-based, open-access platform integrated with a collection of tools that automate the collection, analysis, and comparison of metagenomic data sets. Providing direct access to tools commonly used in the community for analysis of agricultural ecosystem resistomes further supports the ability to address a variety of research questions across projects. In addition to annotation of metagenomes, AgroSeek further supports analysis and comparison of samples and projects based on their metadata attributes. Specifically, AgroSeek aids in streamlining, comparing, and normalizing metadata in a manner that harmonizes analysis across multiple data sets as a function of multiple common attributes, allowing users to compare their projects to other submitted projects. We designed a user-friendly and flexible template format to accommodate user-customized metadata attributes in different environments, with normalized input formats to facilitate sharing of data and projects across the user community to support predictive analysis. Common attributes, such as DNA extraction method and sequencing platform remain mandatory for all samples, given that they are known to introduce bias in sequencing results [15], while others are optional. Furthermore, AgroSeek provides a public demo project to provide a starting place for external comparison of user projects. Insights gained, including results within and across user projects, aid in yielding a comprehensive assessment of the effects of various agricultural practices on metagenomes and actionable information that is informative to policy and practice. Through collective project and data sharing and comparison, we expect that more powerful conclusions can be drawn, within and across projects, to comprehensively address important microbial impacts of agricultural practices, such as the potential to contribute to the spread of ARGs. For example, through tables and figures, users can visualize and assess how their soil, water, or air samples compare to a wealth of other samples submitted by other users. By building a repository for data for comparison and by standardizing the parameters for each tool, we take a substantial step needed to next be able to develop predictive models.

## Implementation

**Fig. 1:**
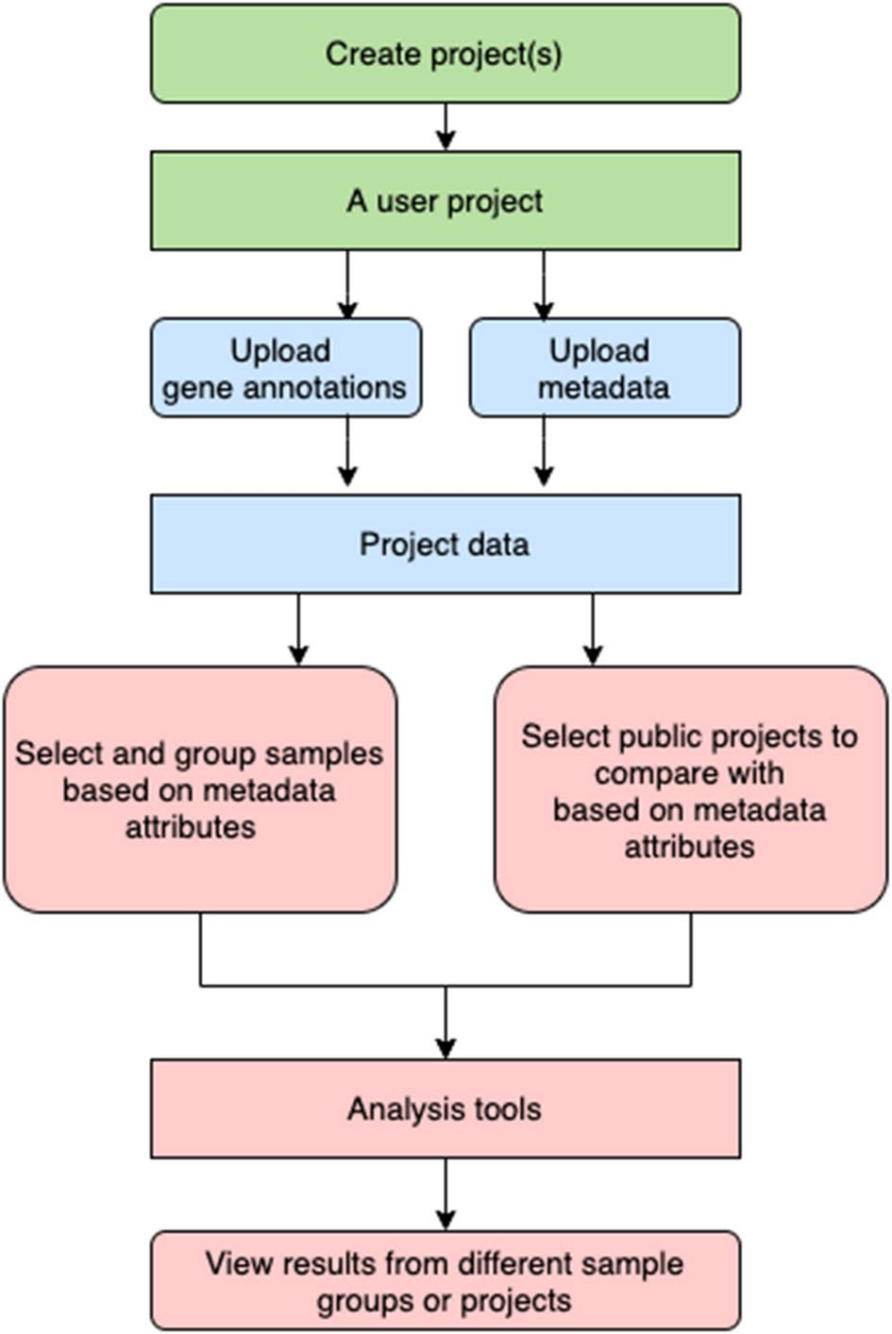
AgroSeek web site major workflow from a user perspective. A user will need to first create a project. After creating a project, the user can upload gene annotations and metadata for this project. The uploaded data will become the “project data” for this project. Project data can be used for analysis after selecting and grouping samples based on their metadata attributes. Comparison to other projects within the AgroSeek community is also available by filtering public projects based on metadata attributes. Groups can also be manually modified after generation. (1) Green phases in the workflow indicate project creation, (2) blue phases indicate data uploading and (3) red phases indicate analysis. Rectangles indicate entities and rounded rectangles indicate user operations

The AgroSeek web site is built upon the WordPress framework and PHP. The data, especially the metadata, are organized in MySQL databases. Analysis tasks are handled using Python and R programs. From a user perspective, the overall workflow is depicted in Fig. 1. A diagram of the web site is available in Additional file 3: Figure S1. On the AgroSeek home page, a tutorial is available for users to walk through workflow examples.

**Fig. 2:**
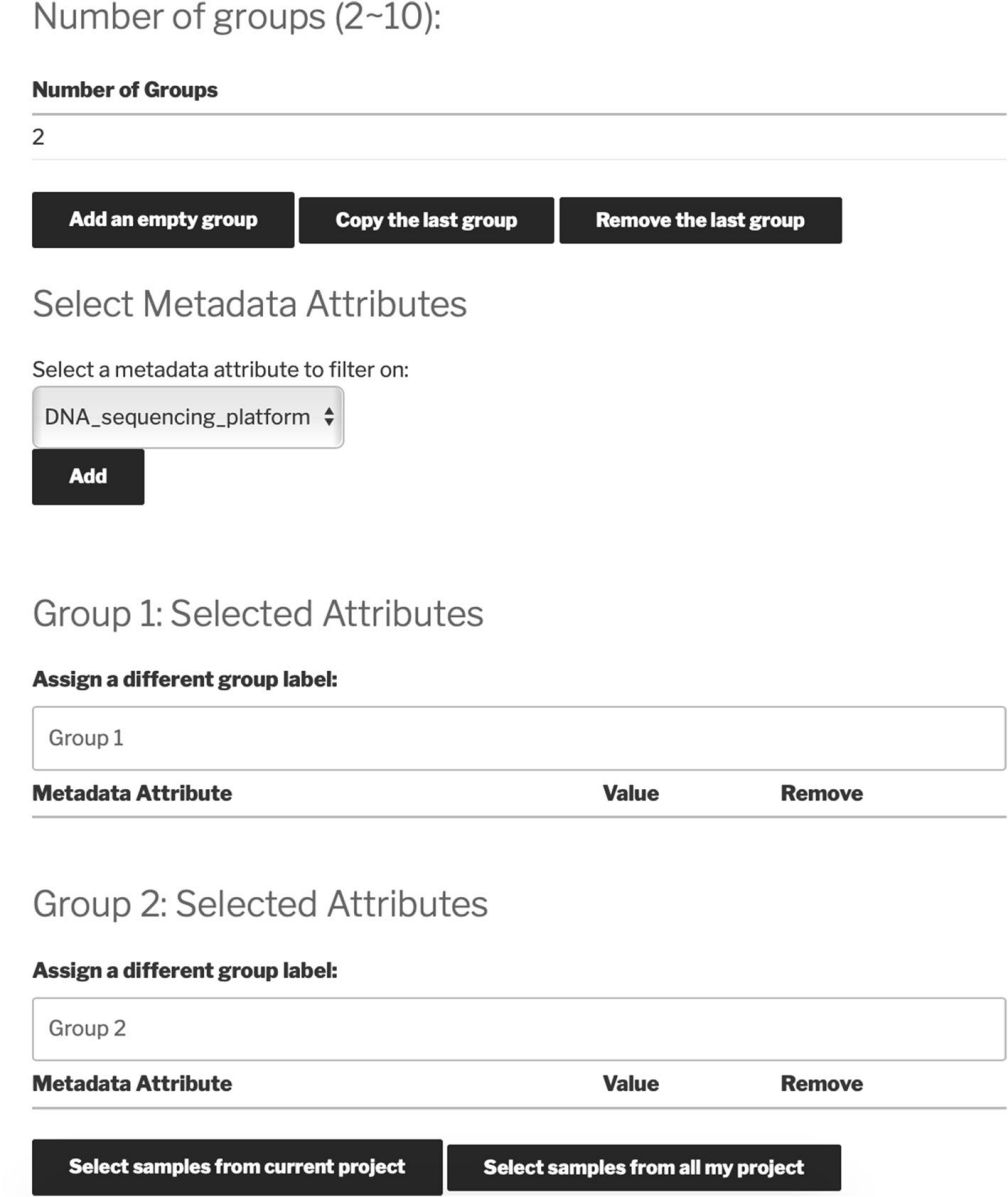
Sample selection and grouping page on AgroSeek. Once the annotations and associated metadata are uploaded to a project, the metadata attributes can be used to filter samples. A file including the filtered samples and assigned groups will be generated after this process, while manual post-modification is also available from a user’s side. Project comparison has a similar process to filter projects containing certain metadata attribute values

### Data uploading

Generally, metagenomic sequences are compared to one or several reference databases to obtain gene annotations. Using annotation tools such as DIAMOND [16] or BLAST, users can annotate genes against the recommended database or their customized databases. Other possible annotation platforms include MetaStorm [14]. A reference database provides sequences of a specific group of genes of interest, such as ARGs. The annotation tool then compares the metagenomic sequences against a reference database to find matches and annotate genes based on the selected annotation threshold values. In the current version, AgroSeek hosts integrated tools that focus on processing relative abundance for each annotated gene.

AgroSeek requires gene annotations processed based on a specified antibiotic resistance reference database (currently, the Comprehensive Antibiotic Resistance Database (CARD) v. 3.0.8 [17]) and a set of recommended parameters, such as the e-value cutoff. The purpose of recommending reference databases and parameters for annotations is to provide relevant and comparable information when comparing different samples, encouraging consistent default parameters to support valid comparisons of samples across different publicly-shared projects, which is a key prerequisite for collaboration. This recommended reference database will be updated periodically to keep current with developments in the field. Information about the reference database and parameters are highlighted on the gene annotation upload page.

In parallel with upload of annotations, users are asked to submit their metadata in a provided metadata template. Users can access templates under different categories on the web site relevant to their environment of interest and fill in the template with or without user-customized columns to provide their metadata associated with gene annotations. Both the gene annotations and metadata can be uploaded and managed in user-created projects.

#### User-customized metadata attributes

On the metadata upload page, AgroSeek offers several metadata templates under different environmental categories (soil, manure, crops, water, and air). Each template is designed to be user friendly and includes detailed instructions. We first ask the users to fill in mandatory columns such as sample ID. Several category-specified optional columns are included as examples. Users can fill in some (or none) of these optional columns and ignore others that do not apply to their specific projects. If the users have other metadata attributes that are not listed in the predefined columns, then they are also encouraged to create and fill in custom columns to include more information in their metadata. A screenshot of one of the metadata templates is available in Additional file 3: Figure S2.

In the template instructions, naming rules and several examples are provided for the user-custom metadata attributes. It is strongly recommended that attribute names be descriptive and self-explanatory. Characters should be in lowercase, spaces and special characters should be removed. Consistent units must be specified for any quantitative attributes. The newly uploaded customized metadata attributes are validated by the web service, then confirmed by the user to proceed.

After the associated metadata attributes of the samples are uploaded, they can be used to select and group the samples or projects. In addition to grouping their own projects and samples, users can include samples from other publicly-available studies on the platform. The selected and grouped samples or projects can then be used to perform analysis or comparison. The corresponding sample selection page is shown in Fig. 2.

### Database

We use a MySQL database to store and manage user data. Mandatory information in user projects and data are reflected in database tables as “NOT NULL” fields. Optional fields are not required and can be set to a default value.

Our database design is expandable with arbitrary metadata attributes, enabling users to customize their own metadata attributes. For example, in certain experiments, a user may be interested in air pressure in the environment when collecting samples, but it may not be a default metadata attribute in the provided metadata tablew. In AgroSeek, this user can customize the “air pressure” metadata attribute to handle such situations and include this information when performing downstream analysis. Furthermore, if a user looks at public projects in the community and finds another project with the same “air pressure” attribute, the user can then compare the two projects in terms of the effect of “air pressure” on corresponding metagenomes. Thus, uploading and sharing customized metadata attributes not only provides more flexibility in analyses, but also facilitates interaction between researchers and practitioners, compared to fixed metadata attributes.

### Analysis tools

After uploading the annotations and metadata to a project, users can choose from a collection of tools to analyze, compare or visualize the data. Current available analysis tools on AgroSeek include DirtyGenes [18], ExtrARG [19] and NMDS [20]. In the sections below, we demonstrate these tools using synthetic and environmental data sets.

DirtyGenes [18] is a computational tool developed in R that identifies statistically significant differences between two sample groups. This open source tool was originally applied to detect differences between relative ARG abundances, but can be extended to apply to other forms of data. DirtyGenes is especially proficient for analysis of small data sets, which is a useful attribute for users for obtaining reliable analysis even if only a few samples are identified that are characterized by the attributes of interest.

ExtrARG [19] is an open source tool developed in Python that uses the extremely randomized trees (ERT) algorithm to efficiently extract differentially abundant ARGs from metagenomics samples classified into different groups. This approach is time efficient and avoids bias towards ARGs with high relative abundances. The current version accepts relative gene abundances and user defined group labels (e.g. sample environments) as input.

The NMDS [20] tool is based on the R package “vegan” [21]. It takes all the numeric columns in an uploaded spreadsheet to compute the distances between samples, then projects the distance matrix to a lower dimensional space. Users can choose which distance measurement they want to use, what number of dimensions they want to project to, and which three non-numeric columns (can be repeatedly used) in the spreadsheet to display the projected groups in color, shape and label, respectively. The outputs include a projected plot and a stress value. A lower stress value indicates better ordination of the projection.

When users access a computational tool on the project page, a task will be scheduled to execute the computation. Once the computation task is finished, the task will be marked as “Done” and the analysis result will then be able to be checked.

**Fig. 3:**
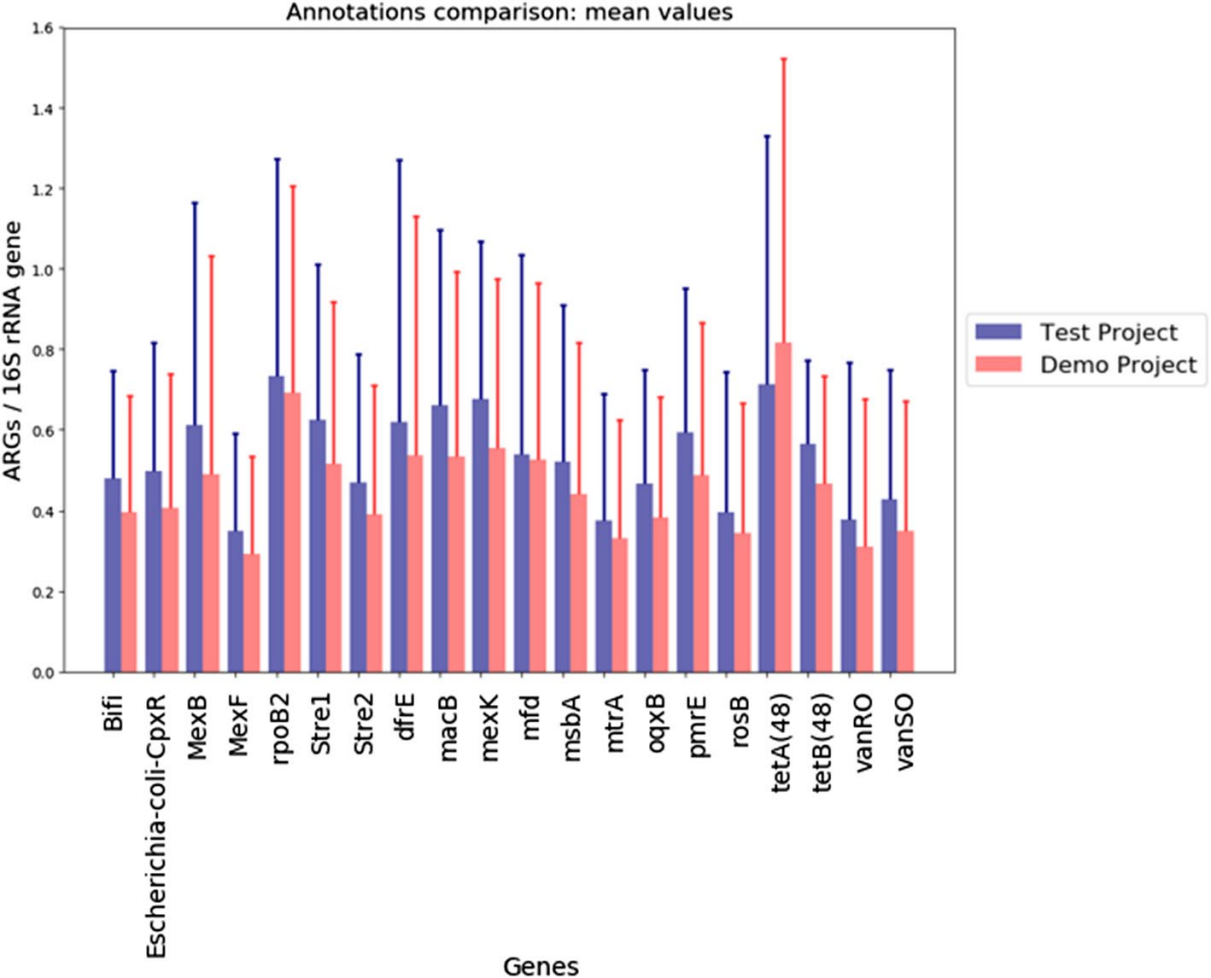
An example of a comparison of the mean values of relative abundances of ARGs between two projects. The project with which to compare the current project can be selected based on their metadata features (e.g. a project with soil type “loamy sand”). The blue bars are from “Test Project”, red bars are from “Demo Project”. Error bars represent standard deviations. The x-axis labels show the antibiotic resistance genes shared by the two projects. Abbreviations for genes: Bifi for Bifidobacteria-intrinsic-ileS-conferring-resistance-to-mupirocin, Stre1 for Streptomyces-cinnamoneus-EF-Tu-mutants-conferring-resistance-to-elfamycin, and Stre2 for Streptomyces-rishiriensis-parY-mutant-conferring-resistance-to-aminocoumarin. The y-axis shows the mean relative abundance for each gene. In this example, both projects contain synthetic data for demo purposes only

Users can also access public projects to conduct inter-project analysis. Currently, we have initiated this functionality with inter-project comparison. Metadata attributes from all available projects will be displayed as filter conditions. Users can choose a range of numbers for quantitative metadata attributes, or an option from the metadata value list to construct a combination of filter conditions. Based on the filter results, users can then choose a filtered out project with which to compare their project. Figure 3 shows a project comparison plot.

**Fig. 4:**
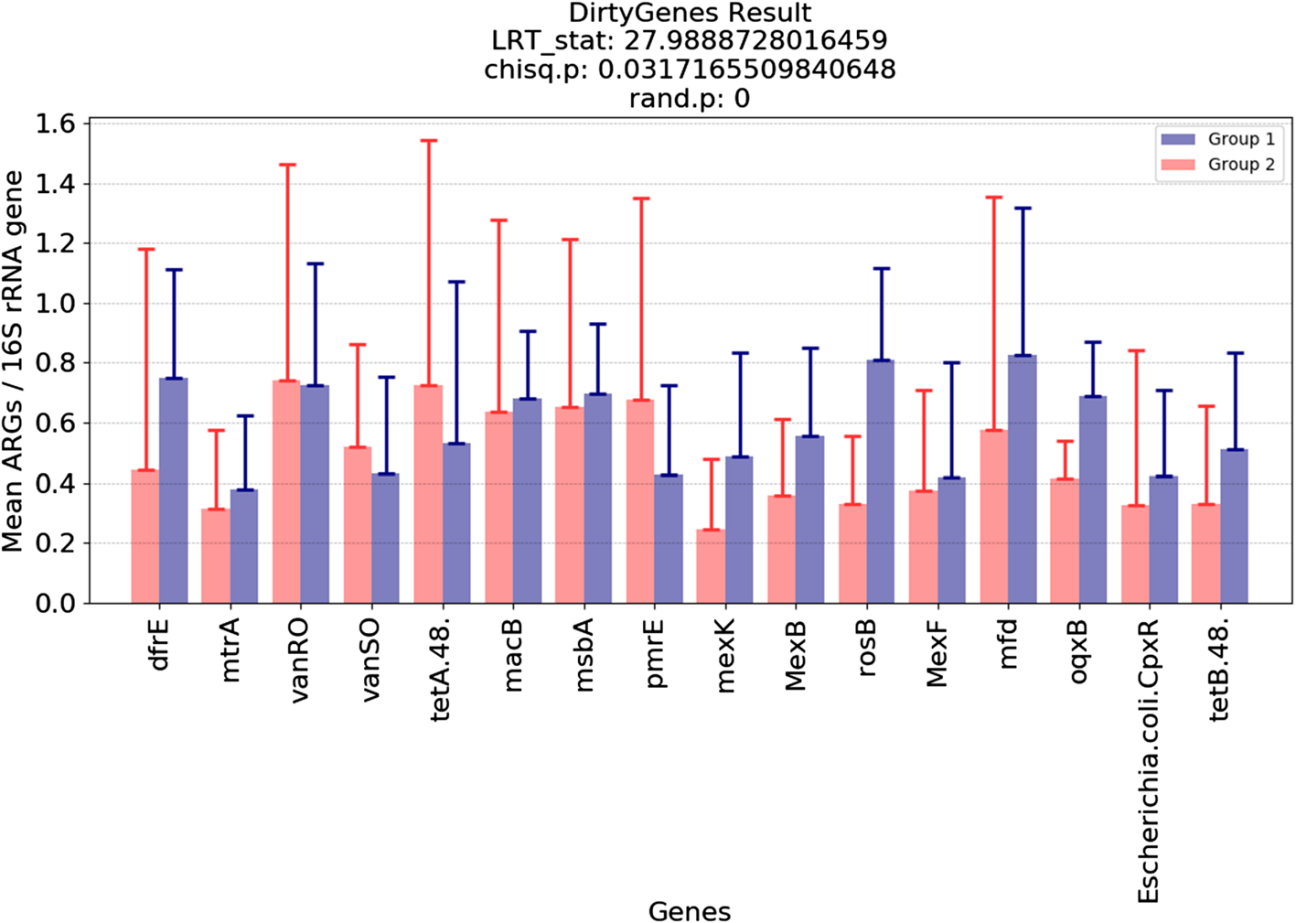
DirtyGenes analysis example. This is a bar plot of the mean values in different groups with indications of DirtyGenes result values. Error bars represent the standard deviations. Groups for different samples can be manually inputted or generated based on their metadata features, as provided as a functionality in our sample selection tool. For this plot, groups are randomly generated. Values of DirtyGene results: (1) LRT.stat is the raw test statistic from performing the likelihood ratio, (2) chisq.p is the *p* value from the chi-squared test, and (3) rand.p is the *p* value from the randomization test

### Data and results availability

All results and analyses can be deleted by the project owner or remain permanently accessible on the project page under “Analysis Results” (see the user interface in Additional file 3: Figure S3). For public projects, project data analysis results can be viewed by registered users, but cannot be edited or deleted.

## Results

### Analysis tools

We have demonstrated the functionality of all three metagenomic data analysis tools currently incorporated in AgroSeek using both real environmental sequencing data and synthetic data randomly generated by a Python script. For DirtyGenes and ExtrARG, we uniformly generated 11 synthetic sample data points within the range [0.01, 2.0) and divided them into two groups: Group 1 and Group 2. For NMDS analysis, we similarly generated 88 sample data points for two groups: Group 1 and Group 2, while 22 samples in Group 1 were generated within the range [0.01, 1.0) and 66 samples in Group 2 within the range [0.03, 2.0) (see the script in Additional file 2). Environmental data and metadata from water and agricultural ecosystems, including wastewater, soil, manure-derived compost, and vegetables grown in the greenhouse and postharvest, were used to assess the flexibility required in metadata comparisons across environments as well as in DirtyGenes, ExtrARG, and NMDS analyses. On the web site, we provide a demo project to access a small portion of the synthetic testing data as well as analysis results. The project is named “Demo Project” under public projects, visible to all logged in users.

**Fig. 5:**
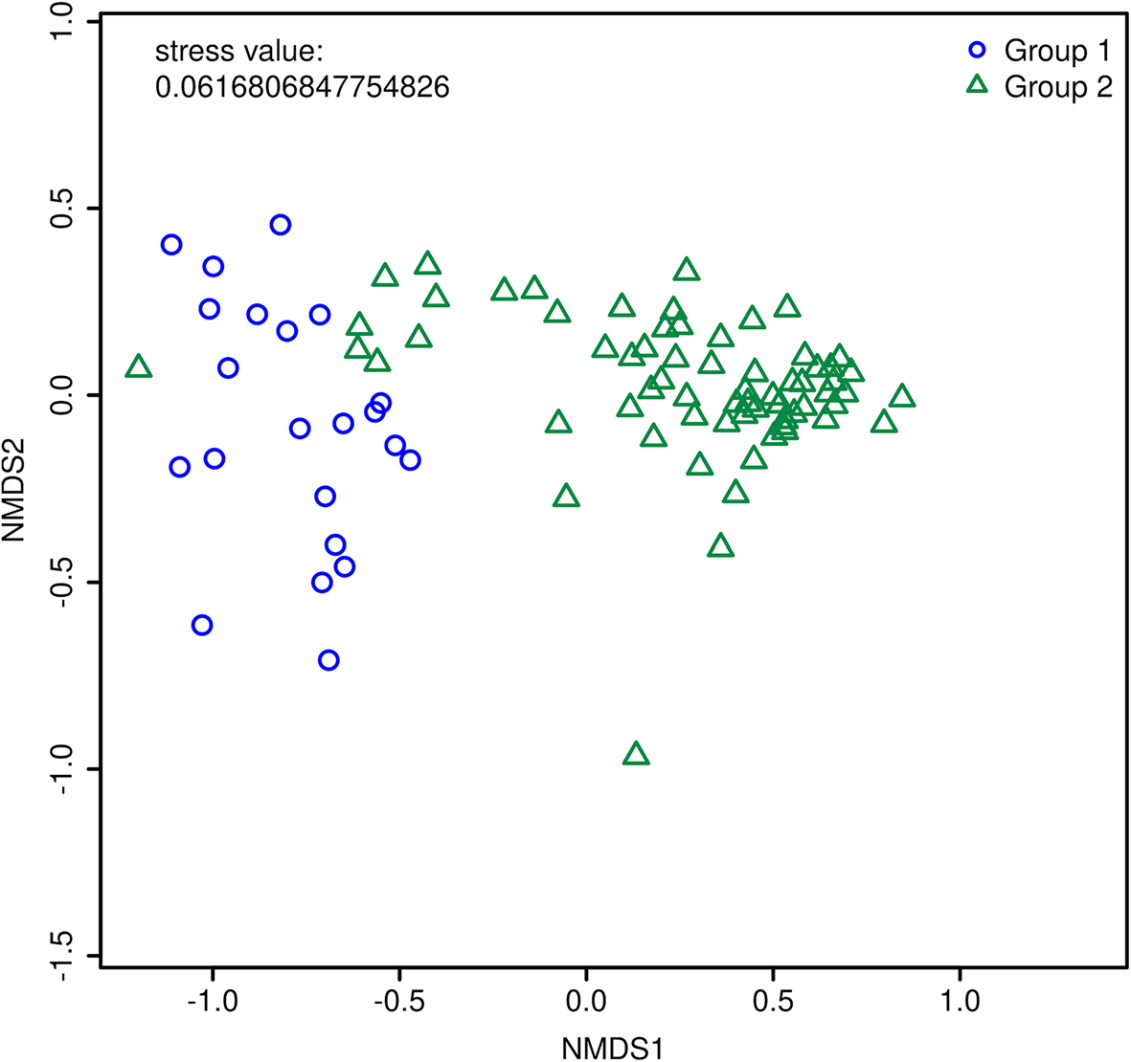
An example of NMDS analysis results generated from synthetic data. The distances between samples are projected to a 2D plot. The axes of a NMDS plot is meaningless and ignorable. Samples are colored based on their assigned groups. This figure shows the distances between and clusters of input samples. Groups for different samples can be manually inputted or generated based on their metadata features, as provided as a functionality in our sample selection tool. For this plot, groups are manually assigned

**Fig. 6:**
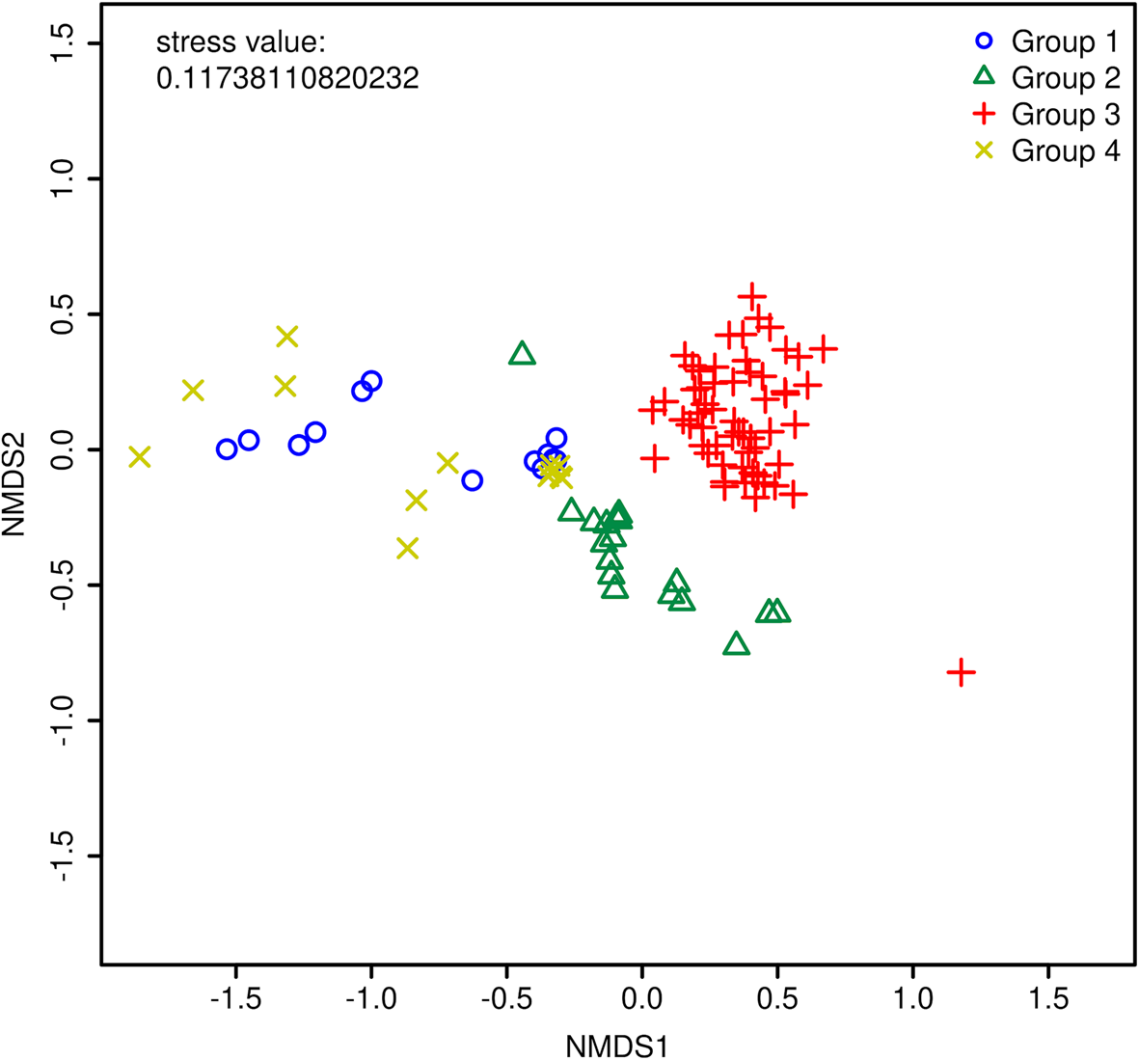
An example of NMDS analysis results generated from a subset of 198 metagenomic samples, in total 101 samples. The distances between samples are projected to a 2D plot. The axes of a NMDS plot is meaningless and ignorable. Samples are colored based on their assigned groups. Groups for different samples are generated based on their metadata features. Group 1: animal_type “Dairy” and treatment “Organic compost”. Group 2: soil_type “Loamy Sand” and time_day_after_amendment “120.0”. Group 3: vegetable_type “Radish”. Group 4: animal_type “Beef”

Figures and tables can be generated from the analysis tools to help users visualize the results. In addition to generating figures on our platform, users have the ability to download generated tables, so that they may customize visualization of their results. Example figures from the platform representing DirtyGenes (Fig. 4), ExtraARG (Additional file 3: Figure S4), and a Bray-Curtis similarity matrix on an NMDS plot (Fig. 5) are shown. Figure 6 is a plot generated from 101 actual shotgun metagenomic sequencing libraries obtained across 3 different environmental media (vegetable, soil, and compost). Groups for the synthetic data are randomly generated except for NMDS analysis. Groups for the 101 real samples are generated based on following metadata features: animal_type, soil_type, vegetable_type, time_day_after_amendment and treatment.

These test runs demonstrate that AgroSeek is capable of handling arbitrary metadata attributes, performing analysis on selected data tables, and retrieving analysis results whenever the user desires to revisit them. These analyses performed on real environmental sequencing data in practice can be used to visually cluster samples into different groups and identify the specific genes that contribute to the differences. For example, DirtyGenes can help understand if two groups are actually from different distributions. NMDS can show how similar the samples in the same group are in terms of resistome. ExtrARG reveals the discriminatory genes and illustrates how much each gene contributes to the difference.

A prototype of AgroSeek was introduced at the U.S. Department of Agriculture funded Human Dimensions of Antimicrobial Resistance in Agriculture Workshop in 2019. This served to obtain feedback from target end-users, including researchers, producers, extension specialists, and veterinarians. In general, attendees saw utility in a publicly-accessible platform that analyzes metagenomic data to help advance policy initiatives. The potential for analysis tools to compare metagenomics samples across studies was particularly valued.

### Comparison to the state-of-the-art

We further compared AgroSeek to alternative metagenomic data analysis platforms, namely MG-RAST [13] and MetaStorm [14] (Table 1). MG-RAST pioneered a platform that offers a user-friendly means to annotate taxonomic and functional genes in metagenomic data sets, while also encouraging publically-available data sharing. However, MG-RAST is not suitable for antibiotic resistance research because it does not interface with current ARG databases. AgroSeek, on the other hand, is specifically tailored for the purpose of tracking ARGs and the contributing factors associated with their occurrence patterns. While MG-RAST provides a detailed report of sequence annotations, the AgroSeek annotation algorithm has been optimized for identification of ARGs, while also leveraging associated metadata to support comparative analysis across projects.

MetaStorm’s main strength is as a user-friendly means to annotate metagenomes using customized databases. However, MetaStorm is not configured to facilitate comparison across different projects . Moreover, although MetaStorm provides a metadata template for sample submission, the template is not tailored to the study of antibiotic resistance and it is not possible to actually draw from the metadata as a factor in the data analysis. AgroSeek provides a means to comprehensively gather relevant metadata in a consistent format (e.g., common units) so that they can be effectively leveraged for analysis and comparison across projects. While the present version is tailored for a user community in the agriculture sector focused on antibiotic resistance, it is anticipated that additional data analysis tools will be incorporated into future versions, along with means for users to tap into a growing database of publicly-available user-provided metadata to expand the capacity of research questions and comparisons that can be made.

The current version of AgroSeek suggests common databases and parameters for annotation as a means of facilitating the ability to compare across projects. To further support comparability across projects in the future, specific databases and parameters will be more seamlessly built into the AgroSeek platform in the future. While users can adjust these databases and parameters and criteria if need be, building a default into the system will facilitate the ability to make broad comparisons. Finally, we emphasize that the AgroSeek metadata templates are flexible in terms of accommodating custom user attributes. Instead of providing a fixed set of general attributes, we provide a limited set of mandatory attributes, along with the ability to incorporate an extensive list of optional attributes in the metadata templates. Moreover, the default metadata attributes and pipeline are fine-tuned with respect to the topic and audience of interest, namely antibiotic resistance in agricultural ecosystems, to conveniently obtain relevant analysis with the first pass.

**Table 1:** A comparison of the MG-RAST, MetaStorm and AgroSeek platforms

	AgroSeek	MG-RAST	MetaStorm
Database	CARD (ARG specific)	General protein taxonomic and classification databases	User customizable databases
Studied metagenomic genes and audience	Specifically tailored to the study of ARGs	General taxonomic and functional annotation audience in metagenomics area, not well-calibrated for ARG studies	Customizable databases that users can choose what to annotate for (currently includes bacterial metals resistance genes, ARGs, and MGEs)
Categories of metadata templates	Subtemplates customized for different environments, one subtemplate for one project	One general comprehensive template and a few subtemplates depending on the project type	A single template for all data types with no interaction within the analysis process
Mandatory metadata attributes	5	$> 33$	None
Inter-reference between metadata attributes	Handled on the platform side	Some need to be handled on user side	None

## Conclusion

AgroSeek provides a new and powerful platform for comparative and, in the future, predictive computational analysis of environmentally-derived metagenomic sequencing data. Foundational to AgroSeek is the inclusion of rich user-provided metadata, which is uniformly formatted and archived to support comparative analysis within and across individual projects. Importantly, making data and metadata publicly available via AgroSeek supports large, systems-scale research to detangle multiple factors driving complex environmental phenomena.

A novel and powerful attribute of AgroSeek is its capacity to gather and normalize metagenomic data and metadata and to facilitate data sharing, analysis, and collaboration across multiple projects. Lack of sufficient data and metadata is a barrier to further computational modeling and analysis. For example, many machine learning models require a minimum data threshold to capture the distribution of the possible classes or groups [22, 23]. The collection of corresponding metadata allows users to normalize and compare data across a range of factors and potential covariates.

This initial launch of AgroSeek and associated metadata collection tools is specifically tailored for tracking ARGs in agricultural ecosystems. The approach can help identify broader patterns in the factors at play in the spread of antibiotic resistance on farms. It facilitates the evaluation of critical control points that can inform effective mitigation practices (e.g., antibiotic use, and manure management). Initial modules have also been developed for the analysis of metagenomes characteristic of various water systems (e.g., surface water, wastewater, drinking water, and recycled water). While we have demonstrated AgroSeek with a set of test data sets and metagenomic analysis tools, this set of tools can easily be expanded in the future. By incorporating additional external and in-house tools currently under development, AgroSeek will be a valuable user-friendly resource that will expand the metagenomic analysis capacity for a variety of users, including researchers, government agencies, NGOs, and others interested in environmental metagenomics as DNA sequencing analysis becomes more economical and accessible. The more that users contribute data to Agroseek, the more value that it will provide to the user community, enabling not only broad multi-project comparisons, but also supporting the potential for predictive functionalities in the future.

### Availability and requirements

- Project name: AgroSeek
- Project home page: https://agroseek.cs.vt.edu/
- Operating system(s): Platform independent
- Programming language: Not applicable
- Other requirements: Web browsers
- License: Not applicable
- Any restrictions to use by non-academics: Freely available to non-academics

## Supplementary Information

**Additional file 1:** Greenhouse metadata template. This is one of the metadata template files provided to the users to customize and upload their sample metadata. This particular template is intended to be used for greenhouse studies. An instruction is included as the first sheet of the spreadsheet.

**Additional file 2:** Synthetic data generation script. This script generates the synthetic data we used as one set of our testing data within specified value ranges.

**Additional file 3:** Supplementary Figures.

## Data Availability

The AgroSeek web Site and data used in the demo project are publicly available at https://agroseek.cs.vt.edu/.

## Abbreviations

ARGs: Antibiotic resistance genes
NGS: Next-generation DNA sequencing
ERT: Extremely randomized trees
NGOs: Non-governmental organizations
