# Supplementary material for: AgroSeek: a system for computational analysis of environmental metagenomic data and associated metadata
Source: BMC Bioinformatics. 2021 Mar 10;22:117. doi: 10.1186/s12859-021-04035-5 (PMC7944603; doi:10.1186/s12859-021-04035-5)
Supplement: Supplementary file 3 — Additional file 3: Supplementary Figures. [file 12859_2021_4035_MOESM3_ESM.docx]

Supplementary figures


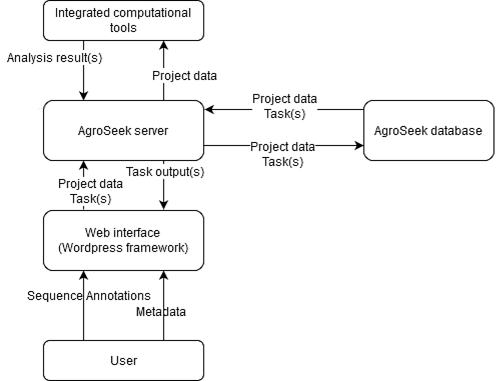


Figure S1 AgroSeek web site diagram. The web interface accepts gene annotations and associated metadata from the user and combines the two as “project data”. Project data is stored in a database via AgroSeek server. When a user schedules an analysis task, the server records the analysis job in a database and uses the retrieved project data to call the integrated computational tools. After the tools send back the analysis results, the server records the results in a database and sends them back to the web interface. If the user later wants to revisit the analysis results, the web interface will talk to the server to retrieve the analysis results from the database.


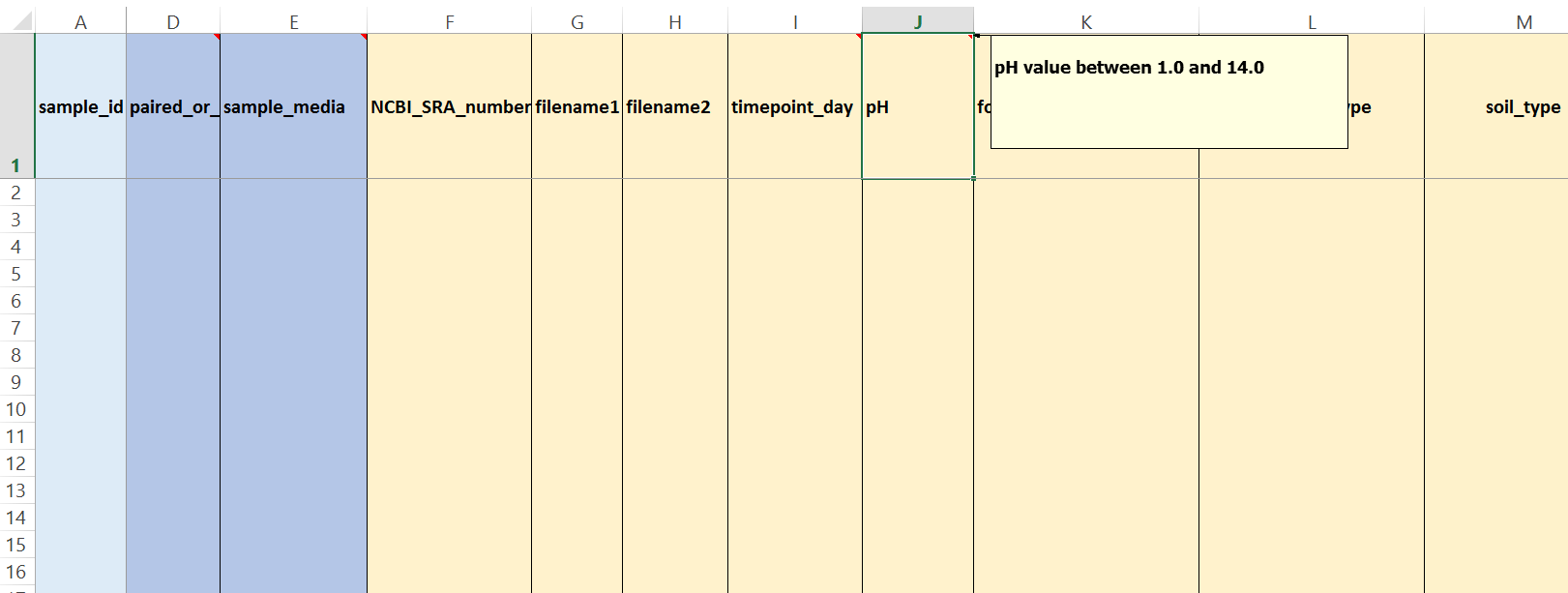


Figure S2 A screenshot of one of the metadata templates. This template is for crops and post-harvest environmental samples.Blue columns are mandatory and yellow columns are optional. Hovering on the column names will display the description of the column.


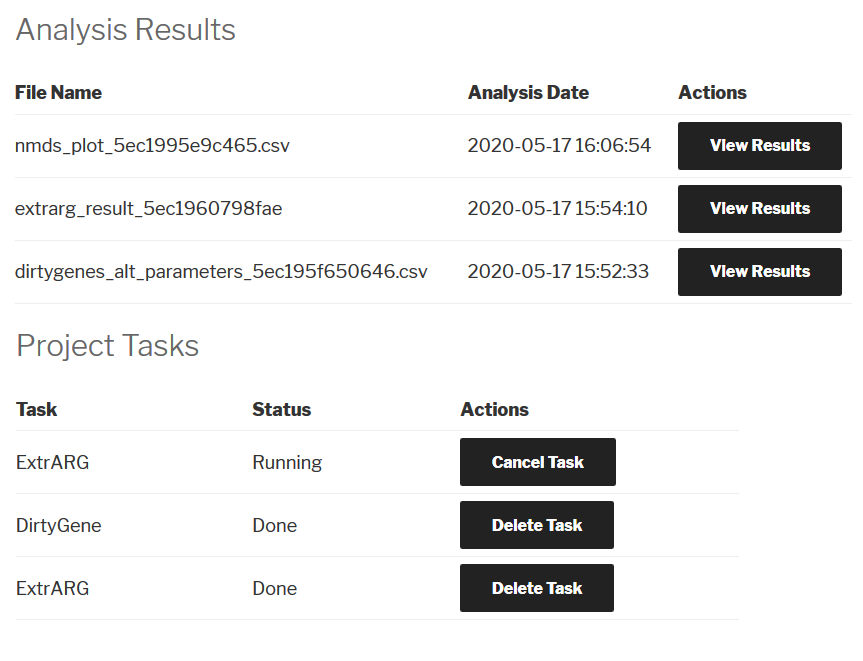


Figure S3 An example of analysis tasks. The web page shows the status of projects and associated results. This web page also shows the analysis result file name, analysis date and options to view the results. If the project is owned by the current user, the user can also delete an analysis result.The status of the tasks can be tracked by the user.


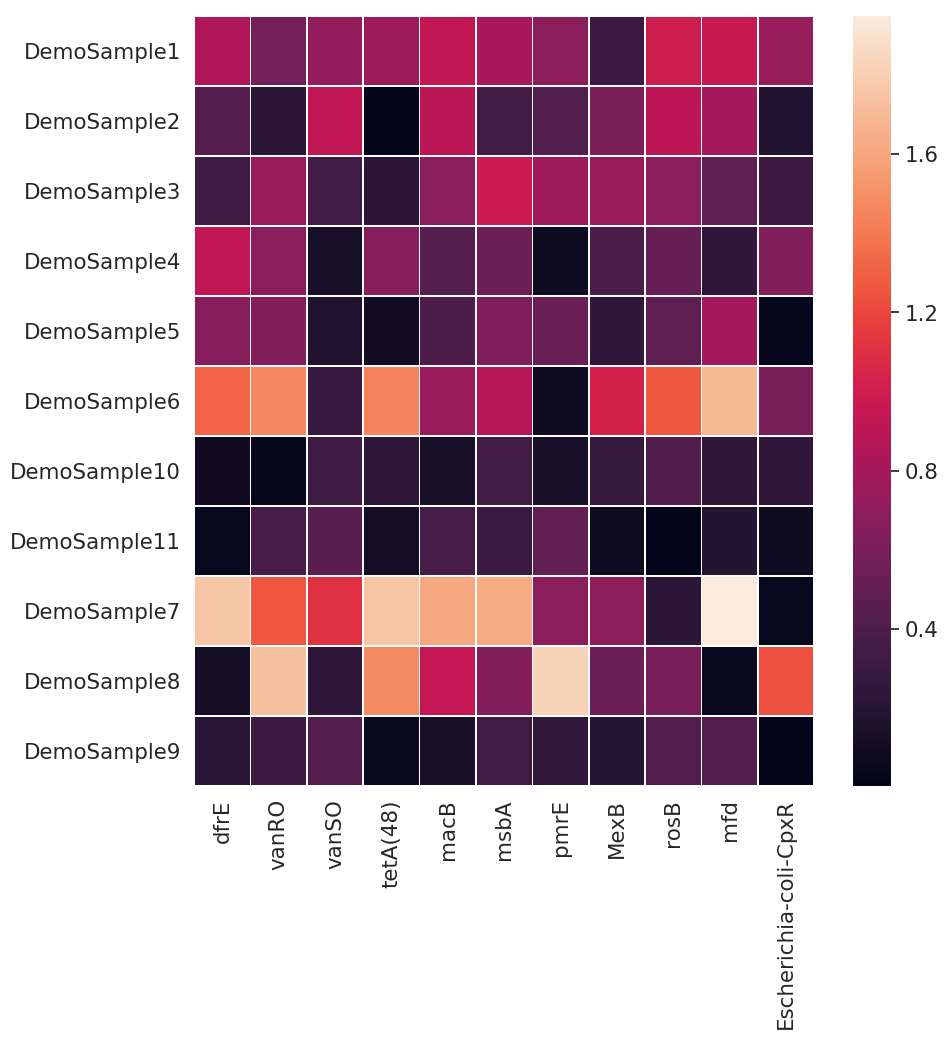


Figure S4 An example of ExtrARG analysis results. This is an example heatmap users can obtain from the full output spreadsheet. The x-axis labels show the discriminatory genes extracted by the ExtrARG tool. The y-axis labels show the samples. This kind of heatmap generated on ExtrARG results helps the users understand how the samples can be distinguished from each other in terms of gene groups. Groups for different samples can be manually inputted or generated based on their metadata features, as provided as a functionality in our sample selection tool. For this plot, groups are randomly generated.
